# Innate and learned components of egg recognition in the ant *Camponotus floridanus*

**DOI:** 10.1098/rsos.231837

**Published:** 2024-06-26

**Authors:** Dani Moore, Juergen Liebig

**Affiliations:** ^1^ School of Life Sciences, Arizona State University, Tempe, AZ 85287, USA

**Keywords:** cuticular hydrocarbons, nestmate recognition, template formation, egg discrimination, queen pheromone

## Abstract

Insect societies discriminate against foreigners to avoid exploitation. In ants, helper workers only accept individuals with the familiar chemical cues of their colony. Similarly, unfamiliar eggs may get rejected at their first appearance in the nest. We investigated egg acceptance mechanisms by introducing different types of foreign eggs into worker groups of the ant *Camponotus floridanus*. Workers from established colonies familiar with queen-laid eggs always accepted eggs from highly fecund queens, but worker-laid eggs only after exposure for several weeks. Workers naive to eggs only rejected worker-laid eggs once they had prior exposure to eggs laid by highly fecund queens, suggesting that prior exposure to such eggs is necessary for discrimination. The general acceptance of eggs from highly fecund queens, irrespective of previous worker egg exposure, suggests an innate response to the queen pheromone these eggs carry. Workers learned to accept queen-laid eggs from different species, indicating high flexibility in learning egg-recognition cues. In incipient colonies with queen-laid eggs that carry a weak queen pheromone, worker-laid eggs were more likely to get accepted than queen-laid eggs from a different species, suggesting that the similarity of egg-recognition cues between the two types of *C. floridanus* eggs increases acceptance.

## 1. Introduction

Social groups such as colonies of social insects are prone to parasitism and exploitation [1]. Recognition of colony members or nestmates is thus essential to protect the colony from the intrusion of foreign individuals [2,3]. Distinguishing foreign from colony-own is accomplished by associating chemical recognition cues with nestmates. We transfer this concept of the learning of recognition cues to the context of brood recognition and assess the interference of chemical signals.

Efficient brood care is essential for the fitness of colony members [4]. This requires that brood recognition and discrimination mechanisms are in place [5]. Similar to the recognition of nestmates, eggs, larvae and pupa are primarily recognized based on chemical cues. Besides the presence of a larvae pheromone, there is little evidence for larvae pheromones in other species [5]. Eggs, on the other hand, can carry chemical signals from the egg layer [6–8], suggesting the presence of two mechanisms of egg recognition based on either chemical cues or chemical signals.

In olfaction, signals are represented by pheromones which fundamentally differ from chemical cues. Pheromones only exist because a sender emits chemicals to inform a receiver while both sender and receiver benefit [9,10]. Cues, on the other hand, are chemicals emitted by an individual in a non-communicative context, such as a metabolic by-product, but that are nevertheless used by others to extract information about the emitter [11].

One of the major evolutionary pathways of pheromone evolution is represented by the sender–precursor model, which states that signals evolve from cues [12]. If the emitter benefits from the receiver response, the emitted cue may be selected to become a more informative and reliable signal. If the receiver benefits from the perception of the chemical cue or evolved pheromone, the receiver is selected to better perceive the cue and refine its response. In the end, the receiver will be able to appropriately respond to the pheromone without prior exposure, i.e. the response is hardwired or innate [10]. Even though responses to pheromones are hardwired, they can nevertheless be changed by learning [13–15]. Nevertheless, the response to pheromones is mostly innate, while the response to chemical cues is often learned [16].

Pheromones are used in many animals in many different contexts such as mate recognition [9]. In social insects, especially ants, pheromones are a major means of organizing their societies [11]. For example, they are used to recruit colony members to food sources, inform about the danger to the colony through alarm pheromones, or regulate reproduction within a colony. The regulation of reproduction is part of the reproductive division of labour, which is a defining feature of eusocial insects [17]. In ants, usually functional queens lay eggs while workers help raise the eggs to adults. Even though workers often retain reproductive abilities, they do not usually lay eggs when a reproductive queen is present in the colony. These queens communicate their presence and fertility status through queen pheromones or fertility signals, which let workers refrain from activating their ovaries [18–21].

In ants and other social insects, chemical cues are essential for nestmate recognition. Members of a colony develop a neural template of chemical recognition cues associated with their colony and then determine if the recognition cues of an individual match their neural template [2]. Matching leads to acceptance while a mismatch leads to rejection. The main components of these recognition cues are cuticular hydrocarbons (e.g. [22]).

In ants and other eusocial insects, cuticular hydrocarbons (CHCs) are not only used for nestmate recognition but can also represent queen pheromones [6,7,23–25]. These hydrocarbons are part of the lipid layer that covers the cuticle of insects to prevent desiccation and are produced by oenocytes below the cuticle [26]. The hydrocarbon layer consists of a complex mixture of various long-chained compounds. In ants, the hydrocarbon profile can encode information about fertility status as queen pheromone [20,21,25], but it can also be used as a cue that allows for differentiating colony members from foreigners [2]. Queens also deposit these queen pheromones on their eggs. Reproductive queens can thus indirectly communicate their presence to workers through their eggs, leading to ovarian inhibition in workers even in the absence of the queen, e.g. in the ant *Camponotus floridanus* [6].

The queen pheromone is important for recognition of the origin of eggs as well. Worker-laid eggs do not carry the queen pheromone and are often eaten by colony members to regulate colony reproduction [27]. In *C. floridanus*, workers eat worker-laid eggs in the presence of the queen while they raise queen-laid eggs. This discrimination mechanism is based on differences in the hydrocarbon layer of the egg surfaces but the worker response to these eggs is not constant across the colony life cycle [28,29]. In the earliest colony stages, the hydrocarbons on the surface of queen-laid eggs are almost indistinguishable from those on worker-laid eggs, and workers eat neither queen-laid eggs nor worker-laid eggs [28,29]. As the colony grows, the queen produces eggs coated with a mixture of shorter-chained hydrocarbons not found on worker-laid eggs [6,28]. At this stage, workers eat eggs that lack the shorter-chained hydrocarbons (e.g. worker-laid eggs and eggs laid by queens of incipient colonies) but they nurture eggs laid by queens from established colonies, which bear the shorter-chained hydrocarbons [6,28]. Worker-laid eggs can be rescued from destruction if hydrocarbons resembling those found on the queen-laid eggs are added to their surface ([6], see also [30]). In the final stages of a colony, after the queen dies, workers begin to produce eggs and workers do not destroy worker-laid eggs [6], suggesting an egg acceptance mechanism beyond the innate perception of a queen pheromone.

The plasticity in *C. floridanus* workers’ response to eggs requires a flexible recognition system that changes according to colony context. Such a flexible recognition system is already present for the nestmate recognition template that is updated when necessary (e.g. [31–33]). Nevertheless, nestmate recognition of adult colony members differs from egg recognition as it does not include innate recognition. We hypothesize that *C. floridanus* workers use learned cues like in nestmate recognition in addition to signals with an innate response to recognize which eggs to nurture and which eggs to eat. Specifically, we propose workers learn the profile of the prevailing eggs in their environment and destroy eggs if (i) they have an unfamiliar smell and (ii) lack the distinctive, shorter-chained hydrocarbons typical of eggs laid by queens from established colonies. Learning is a common mechanism animals use to accommodate variation in their environment [16,34]. *Camponotus floridanus* ants already use CHC profiles to identify colony members and discriminate against foreigners in nestmate recognition [35–37]. We hypothesize that a similar mechanism is involved in egg recognition. Unfamiliar eggs will be accepted if workers are familiarized with their surface profiles. If the eggs carry the queen pheromone, no familiarization should be necessary.

## 2. Material and methods

### 2.1. Study species and culturing conditions

*Camponotus floridanus and C. tortuganus* colonies were collected as foundations from the Florida Keys, USA, between 2001 and 2011 and maintained as described in [38]. Worker-laid eggs came from queenless worker groups orphaned more than 60 days before the start of the experiment.

### 2.2. Experiment 1

**Figure 1.**
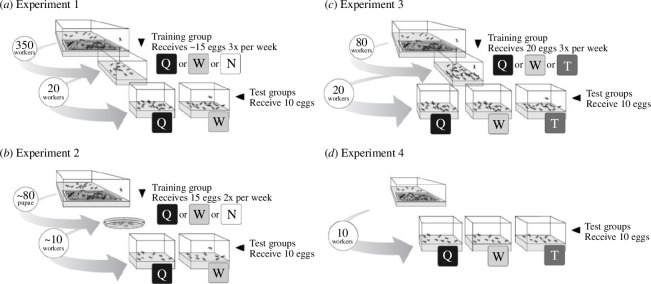
Schematic diagrams showing the experimental design of the four major experiments. Experiments 1–3 use established colonies while experiment 4 uses incipient colonies. In all diagrams, ‘Q’, ‘W’ and ‘T’ denote established *C. floridanus*-queen-laid eggs, *C. floridanus*-worker-laid eggs and *C. tortuganus*-established-queen-laid eggs, respectively. ‘N’ indicates that no eggs were received. Worker selection and egg treatment for (a) experiment 1, (b) experiment 2, (c) experiment 3 and (d) experiment 4. See §§2 and 3 for detailed experimental descriptions.

In the first experiment, we established that workers learn to accept foreign worker-laid eggs once they familiarized themselves with these eggs. Queenless training groups of 350 adult workers were created from 44 established colonies (figure 1*a*). These colonies were 2 to 7 years old and each contained more than 1000 workers. Colonies that did not show the typical pattern of egg discrimination (i.e. colonies in which fewer than five queen-laid eggs survived or more than five worker-laid eggs survived) in week 0 (*n* = 16) were excluded from the study. Excluding these groups was appropriate because we were not interested in characterizing the response of workers to eggs, which has been thoroughly described in other studies [6,28,29]. The purpose of experiment 1 was to test if egg experience is responsible for the change in worker’s response to eggs. Accordingly, we used only colonies that exhibited a strong and unambiguous response in week 0. This selection procedure was not used in any other experiment reported in this study.

Because the workers were isolated from the colony as adults, they had prior experience with queen-laid eggs (throughout the text, ‘queen-laid eggs’ refer to eggs laid by a queen of a large, established colony unless otherwise noted). Each training group was provided with approximately 15 queen-laid eggs (*n* = 15), 15 worker-laid eggs (*n* = 15) or no eggs three times per week (*n* = 14). After 0, 2, 5 and 9 weeks of training, two test groups of 20 workers were isolated from each of the training groups; one test group received 10 worker-laid eggs and the other received 10 queen-laid eggs. In total, 3520 eggs were used for this experiment (see electronic supplementary material S1). After 24 h, we counted the number of surviving eggs in the test groups.

Training groups were maintained in plastic boxes (8 × 10 × 20 cm) with dental-plaster floors and fed ad libitum with water, sugar–water, Bhatkar diet [39] and mealworms.

Training groups were randomly assigned to egg-experience treatment. Training eggs were a mixture from at least three different source colonies. Groups receiving no eggs received larvae to keep pace with the development of larvae in the groups receiving eggs.

For the egg discrimination assay, test groups were placed in a plastic box (8 × 10 × 10 cm) with a moistened, dental-plaster floor and provided test tubes with sugar–water and water. Workers were allowed to acclimatize for at least 30 min before test eggs were gently placed in the colony using a modified Pasteur pipette. After 24 h, we counted the number of eggs remaining.

### 2.3. Experiment 2

In experiment 1, all workers originated from established colonies where they had been exposed to queen-laid eggs. The purpose of our second experiment was to determine if egg experience establishes egg discrimination behaviour in workers without adult experience with eggs (‘naive workers’). Late-stage pupae were collected from 23 large, established colonies. They were placed in plastic Petri dishes (9 × 1.5 cm) with dental-plaster floors and a double-mesh lid together with 20 marked, non-naive workers from twenty-three 3- to 6-year-old colonies. Two days later, any pupae that had not eclosed (i.e. the adult ant worker did not emerge from the cocoon) were removed from the Petri dish, thus creating a cohort of naive workers that eclosed within 48 h of one another. The Petri dish was kept in the parent colony; the double-mesh lid allowed the exchange of volatiles between the experimental group and the parent nest while preventing physical contact. Five days after the uneclosed pupae were returned to the colony, the marked, non-naive workers were removed from the Petri dish, the 5- to 7-day-old naive workers were paint-marked and returned to the Petri dish, and 20 unmarked, non-naive workers were transferred from the parent nest into the Petri dish. All groups received food (Bhatkar diet and half of a chopped mealworm) twice per week, and the unmarked, non-naive workers were exchanged approximately every two weeks.

Training groups were randomly assigned to one of the three treatments. They were provided 15 foreign queen eggs (*n* = 8), 15 foreign worker eggs (*n* = 7), or no eggs two times per week (*n* = 8) (figure 1*b*). The eggs were always a mixture of at least three different colonies. When the workers reached 60 days of age, the age at which workers exhibit egg-eating behaviour (see the following sections), the naive workers were divided into two groups and the response of each group to foreign queen-laid and foreign worker-laid eggs was assayed as in experiment 1. The test groups contained 3–10 individuals, depending on the number of naive workers that survived.

Before the experiment started, we determined at which age workers display egg-destruction behaviour. Single-age cohorts of workers were created following the procedures explained in experiment 2 from each of 38 large, established colonies that were 2 to 10 years old at time of testing. When workers were painted on day 7, they were removed from the experimental Petri dish and returned to the parent nest. Painted workers were collected 15 (*n* = 9), 30 (*n* = 10), 45 (*n* = 10) or 60 (*n* = 9) days after eclosion. Workers’ response to worker-laid eggs was assayed using the egg discrimination assay described in experiment 1. In most cases (*n* = 28), there were enough workers surviving in each colony at the time of testing to use 10 workers in the egg discrimination assays, but fewer workers (3–9, median = 7.5) were used when no more workers were available. Egg discrimination was fully expressed in groups of workers that were 60 days of age; no worker-laid eggs were recovered from these groups. The percentage of worker-laid eggs surviving was 38.9, 29.0 and 27.0 for groups tested at 15, 30 and 45 days of age, respectively.

### 2.4. Experiment 3

To better understand how workers learn to accept eggs with unfamiliar profiles, we performed an experiment with queen-laid eggs from a different species. We were interested in whether recognition cues of eggs that are different from those on *C. floridanus* eggs could be learned by workers.

**Figure 2.**
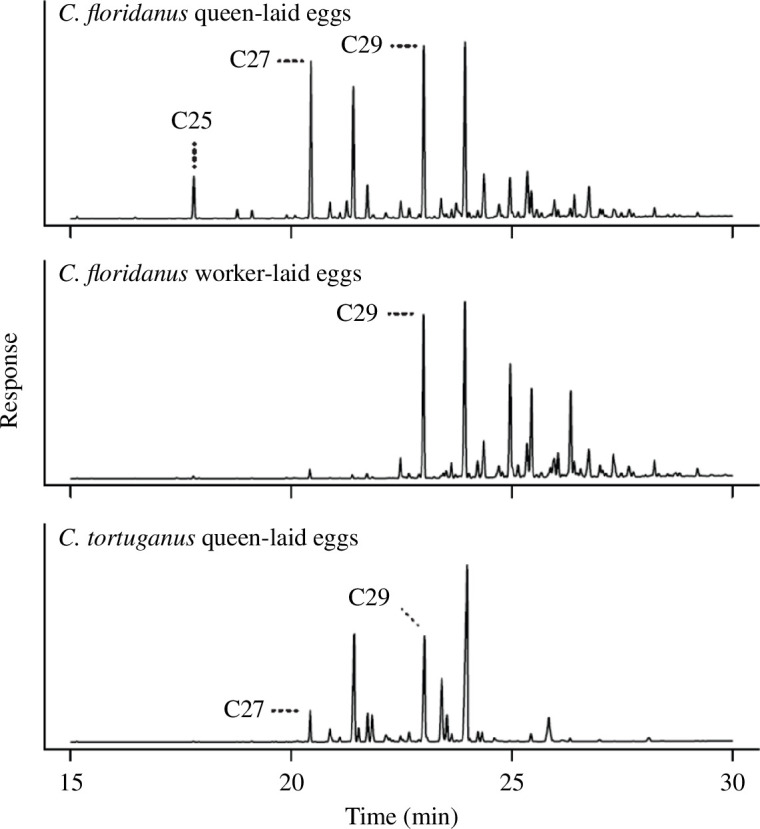
Hydrocarbon profiles of eggs laid by *C. floridanus* workers and queens and *C. tortuganus* queens from established colonies. C25–C29 indicate major alkane peaks (penta-, hepta-, nonacosane).

Groups of 80 minor workers were collected from inside the nest chamber of twenty-four 2- to 5-year-old colonies and maintained as described in Experiment 1. Groups were randomly assigned to one of three treatments in which groups of adult workers with previous experience with queen-laid eggs were trained with foreign *C. floridanus* queen-laid eggs (*n* = 8), foreign *C. floridanus* worker-laid eggs (*n* = 8), or queen-laid eggs from a sympatric species, *C. tortuganus* (*n* = 8) (figure 1*c*). *Camponotus tortuganus* eggs are chemically distinct from both worker- and queen-laid eggs of *C. floridanus* (figure 2). All groups received 20 eggs three times a week. After two weeks, the egg discrimination behaviour of each group was assayed as described above, except workers’ response to *C. tortuganus* queen-laid eggs was tested as well as their response to *C. floridanus* queen-laid and worker-laid eggs.

### 2.5. Experiment 4

To further test the extent of learning involved in egg recognition and acceptance, we tested the response of workers from incipient colonies to *C. tortuganus* queen-laid eggs. Workers from incipient colonies neither eat worker-laid eggs, which are chemically similar to eggs laid by incipient queens, nor established-queen-laid eggs, which are chemically distinct from eggs laid by incipient queens [29]. If this pattern of acceptance arises because workers from incipient colonies have a permissive acceptance threshold or do not destroy eggs, then workers from incipient colonies might accept *C. tortuganus* eggs. If acceptance arises because ants learn to accept eggs with familiar profiles upon repeated encounters, such as eggs laid by queens of incipient colonies, then *C. tortuganus* eggs would not be accepted. Thirty adult workers were removed from 15 incipient colonies, split into three groups of 10 and tested with *C. tortuganus* queen-laid eggs, *C. floridanus* worker-laid eggs or eggs laid by their own queen following the egg discrimination assay described in Experiment 1 (figure 1*d*). This study was conducted using colonies raised from founding queens collected in 2009 when the colonies had between 40 and 75 workers (median = 52).

### 2.6. Gas chromatographic analysis of egg surface hydrocarbons

Chromatograms of hydrocarbons of egg surfaces were obtained by extracting 10 eggs per group in 5 µl hexane for 10 min and injecting 1 µl into an Agilent 6890 gas chromatograph equipped with a flame ionization detector and an Agilent DB-1MS capillary column with 30 m length, 250 µm diameter and 0.25 µm film thickness and an initial flow of 1.0 ml min^−1^ helium. The chromatograph was operated in split/splitless mode with a purge time of 2 min and a temperature of 260°C. The program started with a temperature set to 60°C for 2 min, then it was raised at 40°C min^−1^ to 200°C and continued to raise at 5°C min^−1^ to 320°C, where it was held for another 5 min. The identities of alkanes in the egg chromatograms were determined based on reference chromatograms from gas chromatographic/mass spectrometric analysis using the same column and respective alkane fragmentation patterns and an alkane standard series to identify alkane retention times.

### 2.7. Statistical analysis

Survival of single eggs was analysed using generalized linear mixed models (GLMMs) [40] with binomial error structure and logit link function in experiments 1 and 2. The models were fitted in R [41] with RStudio (2023.06.2) using the function ‘glmer’ of the R package lme4 [42] with training egg type and week and their interaction as fixed factors and colony and time period (only for experiment 1) as random factors. Tests for experiment 1 were done at three different time periods. In Experiment 1, we transformed the factor week for centring. After the model for experiment 1 did not converge, we simplified the model by removing time period as random factor, which led to model convergence. After loading the models, we checked for collinearity by examining the generalized variance-inflation factors (GVIF^1/2df^s) of the general linear models lacking the random effect using the ‘vif’ function of the R package ‘car’ [43]. For experiments 1 and 2, GVIF^1/2df^s were below 2.5. For survival data in experiment 2, we changed one value from 1 to 0 in the worker-egg treatment because all values were 1. By this, we marginally made the original difference smaller. Models were tested against a null model comprising only the random effects structure using a likelihood ratio test. After determining the significance of the full model at *α* = 0.05, we assessed the significance of the interaction term by comparing the full model to a model without the interaction term using a likelihood ratio test for experiments 1 and 2.

The percentage of surviving eggs in each worker group was analysed using a Kruskal–Wallis test with subsequent pairwise comparison for experiment 3 and a Friedman test with subsequent pairwise Wilcoxon matched-pairs test with Bonferroni–Holm correction for experiment 4 using STATISTICA 7.1.

## 3. Results

### 3.1. Experiment 1: does workers’ egg experience affect their response to eggs?

**Figure 3.**
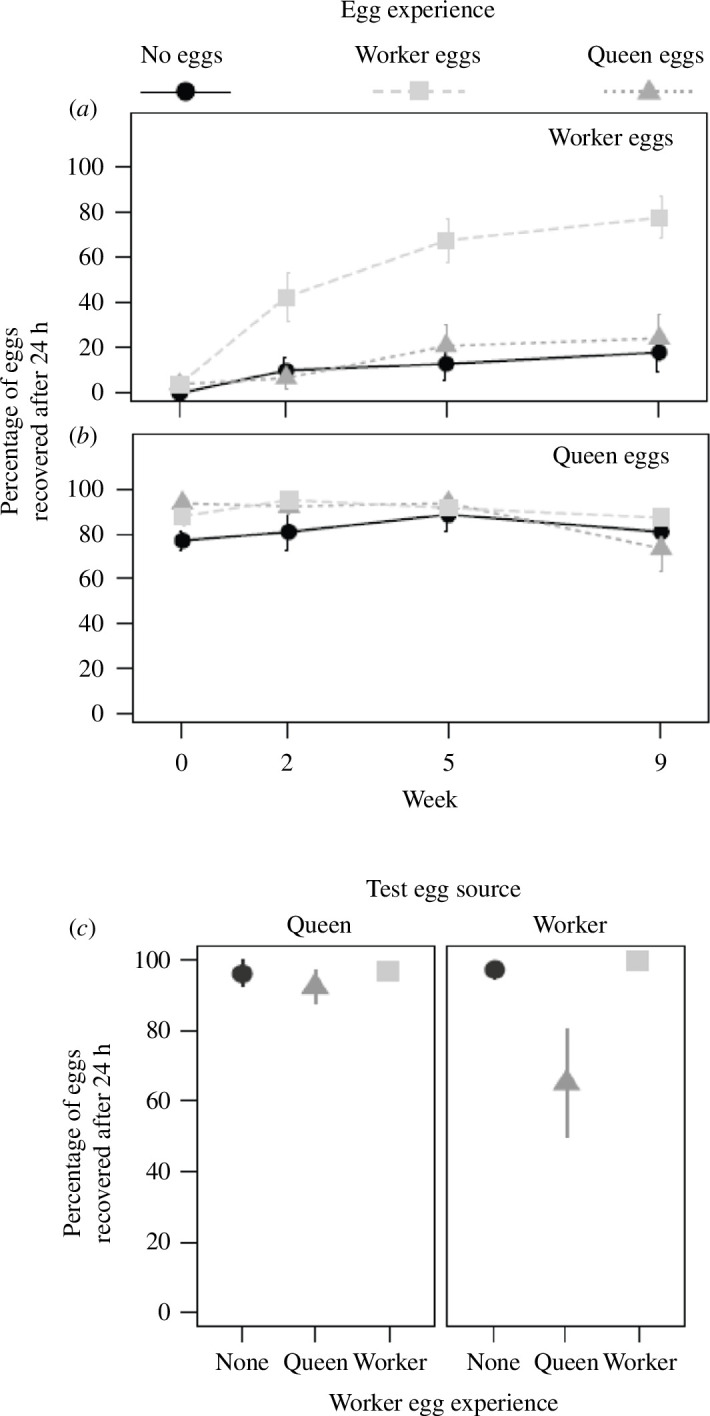
The survival of queen-laid and worker-laid eggs in queenless worker groups with different egg experiences. (*a*) The percentage of worker-laid eggs surviving 24 h after being presented to workers from established colonies tested in queenless worker groups that have experienced queen-laid (triangles), worker-laid (squares) or no eggs (circles) for 0, 2, 5 or 9 weeks. Mean ± s.e., *N* (queen or no eggs) = 15 colonies, *N* (worker-eggs) = 14 colonies (GLMM, *χ* ^2^ = 28.3, *p* = 7.1 × 10^−7^). (*b*) The percentage of queen-laid eggs surviving under the same conditions as in (*a*) (GLMM, *χ* ^2^ = 23.7, *p* = 7.1 × 10^−6^). (*c*) The percentage of queen-laid (left) and worker-laid (right) eggs surviving after 24 h when presented to naive workers from queenless worker groups that have experienced queen-laid (triangles), worker-laid (squares), or no eggs (circles) during the first 60 days of their adult life. *n* = 8, 7, 8 colonies for queen-laid egg, worker-laid egg or no-egg treatment, respectively (GLMM, *χ* ^2^ = 14.8, *p* < 0.0007).

Survival of worker-laid eggs was low across all three treatments at the start of the experiment (4.0%, 4.0% and 0% for queen-egg, worker-egg and no-egg treatments, respectively), but, as predicted, if workers do not eat familiar eggs, survival of worker-laid eggs increased more in groups that were trained with worker-laid eggs (76.7% surviving in week 9) than in groups that received queen-laid eggs (24.0%) or no eggs (17.9%; figure 3*a*) leading to a significant interaction between workers’ egg experience and week of the experiments (GLMM, *χ* ^2^ = 28.3, *p* = 7.1 × 10^−7^).

As expected if queen-laid eggs carry a signal that protects them from destruction, survival of queen-laid eggs was high at the onset of the experiment (94.0%, 88.7% and 78.6% for queen-egg, worker-egg and no-egg treatments, respectively) and remained high after nine weeks for all three treatment groups (75.3%, 88.0% and 82.1% for queen-egg, worker-egg and no-egg treatments, respectively, figure 3*b*). Despite the small change in overall egg survival among the groups, the interaction of workers’ egg experience and the week of experiments was significant (GLMM, *χ* ^2^ = 23.7, *p* = 7.1 × 10^−6^).

The results of Experiment 1 show that the response to worker-laid eggs depends on worker’s egg experience. Only workers that experienced worker-laid eggs increased their acceptance of worker-laid eggs over the course of the experiment. The low survival of worker-laid eggs in test groups of workers that had no egg experience shows that it is not deprivation of queen-laid eggs that causes the change in behaviour, but experience with worker-laid eggs. The high survival of queen-laid eggs across all treatments is consistent with the hypothesis that eggs with queen-specific hydrocarbons are nurtured regardless of workers’ egg experience.

### 3.2. Experiment 2: does egg experience establish workers’ response to eggs in workers with no previous egg experience?

As predicted if eggs with queen-specific hydrocarbons are accepted regardless of workers’ prior experience, survival of queen-laid eggs was high across all treatments (96.3%, 92.5% and 97.1% for no-egg, queen-laid-egg and worker-laid-egg treatments, respectively, figure 3*c*). As predicted if workers eat unfamiliar eggs, the survival of worker-laid eggs was lower in groups that were trained on queen-laid eggs (65%) than in groups that received no eggs or worker-laid eggs (97.5% and 100.0%, respectively), with a significant interaction between workers’ egg experience and test egg source (GLMM, *χ* ^2^ = 14.8, *p* < 0.0007).

Our second experiment shows that *C. floridanus* workers do not eat queen-laid eggs even if the workers have no prior experience with such eggs but with worker-laid eggs. This supports the hypothesis that these eggs bear an innately meaningful signal to which workers respond with nurturing behaviour. Our results are also consistent with the hypothesis that workers discriminate against unfamiliar eggs; workers from groups that previously received queen-laid eggs ate more worker-laid eggs than workers from the other two treatments. The high survival of both queen-laid eggs and worker-laid eggs when presented to workers with no egg experience suggests that workers accept worker-laid eggs unless they had prior experience with queen-laid eggs.

### 3.3. Experiment 3: are ants able to learn egg recognition cues largely different from those of their own species?

**Figure 4.**
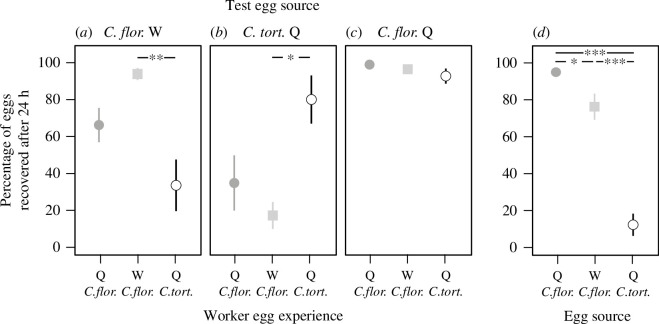
The percentage of *C. floridanus-*queen-laid, *C. floridanus*-worker-laid and *C. tortuganus*-queen-laid eggs surviving after 24 h in queenless worker groups and their hydrocarbon profiles. Eggs were presented to workers that have experienced *C. floridanus*-worker-laid (squares) (*a*), *C. tortuganus*-queen-laid (open circles) (*b*), and *C. floridanus-*queen-laid (filled circle) (*c*) eggs for two weeks. *n* = 8 colonies for each. (*d*) *Camponotus floridanus*-worker-laid, *C. tortuganus*-queen-laid and *C. floridanus*-queen-laid eggs were presented to workers in queenless groups originating from incipient colonies (*n* = 15 each). (Kruskal–Wallis test with subsequent pairwise comparison (*a*–*c*), Friedman test with subsequent Wilcoxon matched-pairs test with Bonferroni–Holm correction (*d*), *p ≤ 0.05, **p ≤ 0.01, ***p ≤ 0.001).

Workers learned to accept eggs that are largely different from queen- and worker-laid eggs of their own species. When compared across the three treatments, eggs had the highest probability of survival when presented to workers that had been trained on the respective egg type (figure 4). *Camponotus floridanus* worker-eggs had a higher survival rate when presented to workers that had experienced *C. floridanus*-worker-laid eggs (93.8%) than workers trained with *C. floridanus*-queen-laid eggs (66.3%) or *C. tortuganus*-queen-laid eggs (33.8%) (Kruskal–Wallis test, H(2, *n* = 24) = 12.4, *p* < 0.003, pairwise comparisons, *C. floridanus* worker-egg versus *C*. *tortuganus* queen eggs, *p* < 0.002, *C*. *floridanus* queen eggs versus *C*. *tortuganus* queen eggs, *p* = 0.071, *C*. *floridanus* queen eggs versus *C*. *tortuganus* queen eggs, *p* = 0.75, figure 4*a*). Similarly, survival of *C. tortuganus*-queen-laid eggs was highest in groups that had experienced *C. tortuganus*-queen-laid eggs (80.0%) followed by groups with *C. floridanus-*queen-laid-egg experience (35.0%) and *C. floridanus*-worker-laid-egg experience (17.5%) (Kruskal–Wallis test, H(2, *n* = 24) = 8.5, *p* = 0.014, pairwise comparisons, *C*. *tortuganus* queen eggs versus *C*. *floridanus* worker eggs, *p* = 0.022, *C*. *tortuganus* queen eggs versus *C*. *floridanus* queen eggs, *p* = 0.11, *C*. *floridanus* queen eggs versus *C*. *floridanus* worker-eggs, *p* = 1.0, figure 4*b*). Consistent with the hypothesis that eggs with *C. floridanus* queen hydrocarbons are always nurtured, survival of *C. floridanus*-queen-laid eggs was high across treatments (98.8%, 96.3% and 92.5% for *C. floridanus-*queen-laid-egg experience, *C. floridanus*-worker-laid-egg experience and *C. tortuganus*-queen-laid-egg experience, respectively) without any significant difference (Kruskal–Wallis test, H(2, *n* = 24) = 1.6, *p* = 0.44, figure 4*c*).

### 3.4. Experiment 4: workers from incipient colonies eat eggs laid by *C. tortuganus* queens

As expected if workers discriminate against unfamiliar eggs, survival of eggs laid by *C. tortuganus* queens was low in our test with workers from incipient colonies (12%) (figure 4*d*). Egg survival was highest among eggs laid by the queen of the test colony (97.3%), followed by *C. floridanus*-worker-laid eggs (78%). Differences among all groups are statistically significant (Friedman test: *χ* ^2^ =24.04, *p* < 0.00001, Wilcoxon matched-pair test: own queen versus worker: *Z* = 2.27, *p* < 0.03, own queen versus *C*. *tortuganus* queen: *Z* = 3.41, *p* < 0.0007, worker versus *C*. *tortuganus* queen: *Z* = 3.30, *p* < 0.001, all comparisons are statistically significant after Bonferroni–Holm correction).

## 4. Discussion

We demonstrated that egg acceptance in *C. floridanus* is based on learned recognition cues and an innate response to queen pheromones if eggs were laid by highly fecund queens. Previously unfamiliar worker-laid eggs and queen-laid eggs from a different species were accepted by workers once they were exposed to these eggs for several weeks. On the other hand, queen-laid eggs that carried a queen pheromone did not require previous exposure to these eggs to get accepted by workers, suggesting an innate response to queen pheromones on queen-laid eggs. Queens of incipient colonies of the size of experimental colonies only show a small fraction of the hydrocarbons specific to the cuticular and egg hydrocarbon profile of highly fecund queens, thus only expressing a weak queen pheromone [28,29]. When queens displayed such weak queen pheromone in incipient colonies, both their own eggs and worker-laid eggs were accepted at high levels, while eggs laid by *C. tortuganus* queens were rejected. Workers accepted unfamiliar eggs that did not carry a queen pheromone when they never had experience with eggs, while such naive workers rejected unfamiliar eggs when they had been exposed to queen-laid eggs previously. These results have important implications for the general mechanism of nestmate recognition and for the interaction of learned egg recognition cues and innate responses to queen pheromones in egg acceptance.

### 4.1. Acceptance of eggs based on learned recognition cues

Workers from established colonies learned to accept unfamiliar worker-laid eggs over the course of several weeks (Experiment 1, figure 3*a*). The ability to learn a chemical profile and discriminate against unfamiliar profiles is well documented in social insects because it is the mechanism used to distinguish nestmates from non-nestmates [2]. Similarly, recognition of ant larvae is based on familiarity in many species [5], and acceptance of eggs laid by non-nestmate queens or by parasitic ant queens is increased by prior exposure to these eggs [44,45]. In many ants, termites and wasps, the chemicals used for nestmate recognition are hydrocarbons [2], the same class of compounds *C. floridanus* workers use to distinguish between queen- and worker-laid eggs [6]. Eggs in many ants differ in their surface hydrocarbons and differences in these hydrocarbon profiles are used for interspecific egg recognition [46,47]. Egg recognition could thus rely on a mechanism similar to the one that allows for nestmate discrimination.

In contrast to unfamiliar worker-laid eggs, queen-laid eggs from foreign colonies were always accepted by workers from established colonies (Experiment 1, figure 3*b*). Such queen-laid eggs from highly fecund queens carry a queen pheromone [6,28,29]. The workers in this experiment originated from established colonies where they had already been exposed to queen-laid eggs. We hypothesize that the presence of the queen pheromone was overriding any potential foreign cues on the queen-laid eggs from foreign colonies similar to the interaction of queen pheromone and nestmate recognition cues in a queen adoption experiment. In that experiment, highly fecund queens from foreign colonies were accepted by workers while weakly fecund queens lacking a strong queen pheromone were rejected [38].

Acceptance of foreign queen-laid eggs is known from honeybees and another ant species [48,49] suggesting that this effect is more common. Similarly, parasitic Cape honeybee workers are naturally successful in depositing eggs in foreign colonies, suggesting an ability to mimic queen-specific cues or signals that allow egg acceptance by workers of the host colony [50]. This is different in the ant *Formica fusca* where foreign queen-laid and worker-laid eggs are destroyed significantly more frequently than worker-laid eggs from the same colony [51]. In this species, social parasitism with the intrusion of foreign queens is frequent and thus more selection pressure on recognition abilities should be present. In the *C. floridanus*, however, parasitism by other ants is unknown and thus we expect selection on foreign egg recognition to be low or absent.

### 4.2. Egg acceptance and the formation of an egg recognition template

Workers in experiment 1 originated from established colonies and were thus already familiar with queen-laid eggs. The use of naive workers without prior egg experience in experiment 2 allowed us to assess the formation of an egg recognition template and if innate responses to queen pheromones are present. Surprisingly, naive workers accepted worker-laid eggs, while in all other experiments, where workers had prior experience with eggs from highly fecund queens, worker-laid eggs were rejected (experiment 2, figure 3*c*). This suggests the presence of an open egg recognition template in workers naive to egg recognition cues. Upon the first encounter of the egg-recognition label represented by the chemical cues associated with the eggs, the workers form an egg-recognition template. This idea is compatible with the result that workers do not accept worker-laid eggs after exposure to queen-laid eggs presumably because they already formed an egg-recognition template based on recognition cues from queen-laid eggs.

This is similar to the model of template formation in nestmate recognition [3]. Social wasps, for example, form a recognition template of their nestmates early in life which is then used for the discrimination of foreigners [52].

An alternative explanation for acceptance of worker-laid eggs without prior experience is that workers use their nestmate recognition template for egg recognition. Egg surface profiles and worker CHC profiles are very similar though not identical [28]. Workers thus might apply a template of nestmate recognition cues to eggs. Whether workers are able to apply such templates to different contexts is, however, unclear.

### 4.3. Acceptance of queen-laid eggs based on innate responses to queen pheromones

Queen-laid eggs were also accepted by naive workers (experiment 2, figure 3*c*). However, queen-laid eggs were also accepted when workers trained with worker-laid eggs were exposed to them for the first time. In this case, queen-laid eggs were accepted even after workers had presumably formed an egg-recognition template based on egg-recognition cues from worker-laid eggs. This suggests that queen-laid eggs carry a queen pheromone that lets workers accept the eggs upon first exposure even when the workers already developed an egg-recognition template based on worker-laid eggs. This is the definition of an innate or hardwired response [10]. Thus, we conclude that acceptance of queen-laid eggs, even though they were foreign, is based on an innate response to the hydrocarbon-based queen pheromone present on queen-laid eggs. Pheromone recognition is generally considered innate or hardwired, and thus does not need to be learned [16]. This applies not only to monomolecular pheromones that are associated with specific neurophysiological adaptations [53] but also to multi-molecular pheromones with additional odour processing in the brain [54]. Although queen pheromone responses are generally considered innate [16], we are not aware of any previous experiment that confirmed an innate response to hydrocarbon-based queen pheromones with naive workers.

We exclude that potential pre-imaginal experience (e.g. [55]) of queen-laid eggs in the larval stage contributed to the acceptance of queen-laid eggs without prior experience of these eggs in naive worker groups (experiment 2). If this would have been the case, workers should have rejected worker-laid eggs in the no-egg treatment based on the potential pre-imaginal experience of queen-laid eggs which was not the case.

### 4.4. Templates of egg recognition cues that are largely different from those of their own species

The previous two experiments included only two kinds of eggs: foreign worker-laid eggs and foreign queen-laid eggs. With eggs laid by queens from a foreign species, we were able to determine that workers could form an egg recognition template from eggs that largely differ in the surface chemicals from those of their own species. Workers always accepted those eggs that they were trained on including the eggs from *C. tortuganus*. Thus, the formation of egg recognition templates is not restricted to eggs from their own species.

### 4.5. Egg recognition templates in incipient colonies with weak queen pheromone presence

Workers in incipient colonies accept both eggs laid by foreign highly fecund queens and eggs laid by foreign workers [29]. We were thus interested if this was due to a highly permissive egg recognition acceptance threshold or if the egg recognition cues of these eggs are similar to the eggs from their own queens that lack most of the queen pheromone components of highly fecund queens and that display a surface hydrocarbon profile similar to worker-laid eggs [29]. While the level of acceptance of queen-laid eggs and foreign worker-laid eggs was different, workers discriminated much more strongly against queen-laid eggs from a different species with a surface hydrocarbon profile that is largely different from queen- and worker-laid eggs (figure 2). This suggests that the similar rates of egg acceptance of queen-laid and foreign worker-laid eggs is not due to the absence of or a permissive egg acceptance threshold, but is due to a similarity of egg recognition cues between the two types of eggs. This is further supported by the fact that incipient *C. floridanus* colonies show nestmate recognition [29].

As discussed above (§4.2), workers may either have an open egg recognition template or apply their nestmate recognition template when they do not have prior egg experience. When young ants first encounter queen-laid eggs in incipient colonies, they associate the egg-recognition cues with those eggs, i.e. form an egg recognition template. The similarity of the egg recognition cues between queen-laid eggs in incipient colonies and worker-laid eggs (e.g. [29]) would explain the high levels of the acceptance of worker-laid eggs even though they did not encounter them previously.

### 4.6. Egg recognition cues and innate pheromone responses

Queen-laid eggs change the composition of their surface hydrocarbons. With increasing colony size and queen fecundity, more components of the queen pheromone appear [6,29]. With this change, workers should be less and less inclined to accept worker-laid eggs because the egg-recognition template they presumably acquire with their first exposure to queen-laid eggs increasingly differs from the label of worker-laid eggs. This is exactly what has been shown with the increase in colony size [29]. This means that workers constantly update their recognition template for queen-laid eggs with an increase in the queen pheromone. Similar effects are known for nestmate recognition where workers update their nestmate recognition template with changing colony odours [3].

Interestingly, the results indicate that egg acceptance of queen-laid eggs is based on two mechanisms, acquired egg recognition templates and innate response to queen pheromones. It is unclear whether these two processes act in parallel or if an innate pheromone response gradually replaces acquired egg recognition templates. A flexible response to varying levels of pheromone seems possible given that innate responses to pheromones can be changed by learning [13–15].

### 4.7. Egg eating and fitness

The eating of worker-laid eggs by other workers in ant colonies with single-mated queens is expected as worker egg laying may reduce colony productivity and thus worker-inclusive fitness, leading to worker egg policing [27]. However, worker egg laying in the presence of a fecund queen in *C. floridanus* colonies is very unlikely. Even in queenless conditions, workers from established colonies did not readily lay eggs but produced their first eggs only after about 60 days without a queen, while between 32% and 58% of queenless worker groups never laid eggs within 160 days, suggesting a high worker egg-laying threshold [6]. If egg laying does not occur in the presence of the queen, even though it may have happened in the ancestors of this species, it is unclear why egg-eating behaviour should be present as a means of policing. An alternative explanation is that egg eating might result from a general rule of thumb to destroy unfamiliar eggs that do not match the known egg template similar to nestmate recognition. Proper identification of colony-specific brood is important as it avoids mistaking the brood as food items. Gradual acceptance in queenless colonies is, on the other hand, expected in both scenarios because the queen no longer produces offspring, and one way of increasing worker direct and indirect fitness is to produce and raise male sexuals originating from workers [27].

## Data Availability

The datasets and code supporting this article have been uploaded as part of the supplementary material [56].
